# Effect of Linseed (*Linum usitatissimum*) Groats-Based Mixed Feed Supplements on Diet Nutrient Digestibility and Blood Parameters of Horses

**DOI:** 10.3390/ani10020272

**Published:** 2020-02-10

**Authors:** Markku Saastamoinen, Susanna Särkijärvi

**Affiliations:** Production Systems, Natural Resources Institute Finland (Luke), FI-31600 Jokioinen, Finland; susanna.sarkijarvi@luke.fi

**Keywords:** feeding, haematology, flax seed, fibre

## Abstract

**Simple Summary:**

In this study, the effect of linseed groat-based fibrous feed supplements on diet digestibility was studied. In addition, possible detrimental health effects due to continuous feeding of such supplemental feeds containing linseed were examined by evaluating blood parameters. The supplemented diets had statistically significantly higher digestibility of crude protein compared to the control diet. In addition, the digestibility of fat (ether extract) was higher in the supplemented diets than in the basal feeding. There were no statistically significant differences or trends in the blood parameters between the treatments. It is concluded that linseed by-products (linseed groats 0.8 g/kg BW/d) combined with other fibre sources can be safely used, for example, in feeding strategies replacing grains in the horses’ rations in order to reduce the intake of starch.

**Abstract:**

Linseed (*Linum usitatissimum*) and its by-products are common supplements used in equine diets and are claimed to have beneficial health effects. In this study, the effect of linseed groat-based fibrous feed supplements on diet digestibility was studied. Also, possible detrimental health effects due to continuous feeding of supplemental feeds containing linseed were examined by evaluating blood parameters. The experimental design was arranged as two balanced 3 × 3 Latin Squares. The horses were individually fed at the maintenance energy level, the forage-to-concentrate ratio being 70:30, with three diets: (A) Control diet consisting of dried hay and whole oats; (B) Control diet + Feed 1; and (C) Control diet + Feed 2. Feed 1 contained 70% of linseed groats, 15% dried carrot, 10% dried garlic and 5% molasses. Feed 2 contained 65% linseed groats, 15% molassed sugar-beet pulp, 10% dried garlic, 5% dried carrot and 5% molasses. Digestibility data were obtained by using chromium mordanted straw as an indigestible external marker for the estimation of apparent digestibility. Blood samples were collected from the jugular vein at the end of each feeding period to evaluate the possible effects of the supplemented diets B and C on the health of the horses. Diets B and C had a higher digestibility of crude protein compared to the control diet A (*p* < 0.05). In addition, the digestibility of ether extract was higher in the supplemented diets than in the basal feeding (*p* < 0.01). There were no statistically significant differences or trends (*p* > 0.05) in the blood parameters between the treatments. It is concluded that linseed groat-based supplements (offering approximately 6.3%–6.7% linseed groats in the diet’s dry matter (DM), or 0.8 g/kg BW/d), and feed containing soluble fibre sources (sugar-beet pulp, dehydrated carrot), improved the crude protein and fat digestibility of hay-oats diets of horses, and can be used, for example, in feeding strategies replacing grains in the horse rations in order to reduce the intake of starch without any adverse effects on the blood parameters and health of the horses.

## 1. Introduction

Linseed (*Linum usitatissimum*) or by-products (groats, cakes, meals) of linseed oil pressing have been used in human and animal nutrition for decades because they are believed to have numerous beneficial effects, many times without any scientific evidence. The “basic” horse nutrition literature in different countries [1,2,3] has recommended the feeding of linseed in various amounts for a long time as a supplemental feed to promote gut and skin health as well as coat quality. Thus, linseed products are commonly used in equine diets [4,5]. However, there is a knowledge gap on the nutritional and health effects of feeding linseed products to horses, because scientific research about this is scarce. For example, proper and safe supplementation levels are not given or known.

Linseed meal is high in crude fibre, acid detergent fibre (ADF) and neutral detergent fibre (NDF) [6]. Pectins and other dietary fibres of linseed have been proved to promote the health of the gastrointestinal tract in humans and dogs (e.g., [7,8]). The hull fraction (outer seed coat) contains 2%–7% polysaccharide mucilage [6,9]. Mucilage is readily water dispersible and forms a viscous slime, which is believed to have positive effects on the stomach and gut [10]. Further, in our preliminary study [11], linseed-based feed enhanced sand removal from the digestive tract of the horses.

Linseed oil is a good source of valuable fatty acids (omega-3) [12]. Groats and meal from cold-pressing may have an oil content of up to 20% [13]. Thus, linseed can be viewed as a cost-effective and economical way to boost omega-3 fatty acids in the feed [14]. In one study [15], a significant improvement in a skin test response to *Culicoides* spp. was reported due to linseed supplementation. Improved hair coat and skin condition scores have also been obtained in dogs after one month of linseed supplementation [16]. Horses have low fatty acid elongation activity, which is important for the inflammatory response, and there is speculation that linseed as a source of omega-3 PUFA may decrease signs of laminitis by inhibiting inflammatory mediators [17]. In a quite recent study [18], increased concentrations of red blood cells, haemoglobin and haematocrit, as well as improved n-3 fatty acid profiles, were reported as a result of linseed oil supplementation. Vineyard et al. [19] found that supplementing horses with milled linseed resulted in pronounced early inflammatory responses to phytohaemagglutinin injections. Both studies also showed increased fatty acid contents of red blood cell membranes. In addition, fats are an important source of energy for horses [20] and can be applied to reduce the starch content of the diet. However, the ether extract digestibility of linseed observed for horses was lower compared to oats and bran [21].

Linseed by-products are rich in protein [6], but a comparison of linseed meal to blended milk products showed that the growth and feed/gain were much better for milk products, the main reason being their better lysine content [22]. Linseed by-products have not been successfully used as protein sources in chicks either [23]. Instead, conflicting results on the effects on growth and health have been reported for pigs [24,25].

Consequently, there are several reasons for the interest in including linseed meal or oily linseed by-products in horse diets by horse owners [5]. However, linseed is known to contain compounds that may be toxic or have anti-nutritive properties [6,26], when the enzyme linase releases cyanide from the glycoside and diclucosides of the seeds [27]. Cyanide levels in linseed are below the level hazardous to humans [6], but there is some concern about the possibility of cyanide poisoning in horses, which are fed linseed [3]. However, intoxications or studies on this matter and where the daily intakes are given have not been reported in horses.

Williams and Lamprecht [28] reviewed studies where linseed oil has been fed to horses, but the feeding of linseed by-products has rarely been the subject of controlled studies with horses, or any other species for that matter. In addition, data regarding the effects (beneficial or detrimental) on diet digestibility and/or animal health when large amounts are fed is scarce. Instead of this, rather small amounts (only 50–120 g/d) are recommended to horse diets [2]. Science-based levels are not given, but recently Lindinger [29] concluded in his article in a veterinary science journal, based on a trade blog [4]), that the highest recommended amount for horses is 454 grams/d (1 pound). Neither research data exists regarding feeds in which linseed is combined with botanically diverse fibre sources.

Therefore, the objectives of this study were to: (1) investigate the effect of two linseed groat-based mixed feed supplements containing other fibrous ingredients on diet digestibility; (2) evaluate the possible detrimental health effects due to continuous feeding of linseed groats supplements in terms of blood parameters. The hypothesis was that there will be no detrimental effects due to the linseed supplementation on the diet digestibility and the haematological values of the horses. The results can be applied in the practical feeding of horses, or by the feed industry utilising linseed groats as a feed ingredient.

## 2. Materials and Methods

### 2.1. Horses and their Management

The influence of two linseed-based feed supplements on diet digestibility was examined with six Finnhorse mares (5–14 years; mean initial BW 636 kg, s.d. 37.8 kg), owned by MTT Agrifood Research Finland (currently Natural Resources Institute Luke). The experiment was conducted in the facilities of Luke. The experimental horses were individually housed in stalls (3 × 3 m) with peat as bedding. The horses had free access to water and a salt block and they were de-wormed before the experiment, and dental care and vaccinations had also been carried out regularly prior to the experiment. During the experiment, they were freely exercised daily in outdoor paddocks for four hours, and one hour by riding at a slow walk, to fulfil their needs of exercise and ensure their wellbeing.

The experimental design was arranged as two balanced 3 × 3 Latin Squares. Each experimental period consisted of 21 days: 16 days of adaptation to the new diet followed by a five-day period of collecting representative spot faecal samples. The BW (electronic animal scale Lahden Vaaka/Lahti Precision Ltd., Lahti, Finland) of the horses was monitored after each collection period to control possible changes and to adjust the individual energy intakes if necessary.

In animal handling and sample collection, the European Union recommendation directives (1999/275/EU) and national animal welfare and ethical legislation set by the Ministry of Agriculture and Forestry of Finland were followed carefully. The experimental procedures were evaluated and approved by The Animal Care Committee of MTT (Permit 9/2001) before the study was started.

### 2.2. Experimental Feeds and Feeding

The horses were randomly allotted to three dietary treatments: (A) Control diet consisting of dried hay dominated by timothy grass and whole oats; (B) Control diet + Feed 1; and (C) Control diet + Feed 2. Feed 1 contained 70% of linseed groats, 15% dried carrot, 10% dried garlic and 5% molasses. Feed 2 contained 65% linseed groats, 15% molassed sugar-beet pulp, 10% dried garlic, 5% dried carrot and 5% molasses. The hay and oats for this experiment were produced by Luke. Feed 1 and Feed 2 were manufactured in a single batch for this experiment by a Finnish medical and food factory (Neomed Ltd., Somero, Finland) and were in granulated form (granulated in 70–80 °C heat for 5 to 6 min). The linseed groats in the experimental feeds were by-products of cold-pressing of linseed oil with an average fat (oil) content of 20%. The other raw materials were included in the experimental feeds in order to improve the palatability as well as owing to their technological properties [30]. They are also common supplemental feeds included in horse diets. The average chemical composition of the feeds is presented in Table 1.

The horses were individually fed at the maintenance energy level according to the Finnish Feed Tables and Feeding Recommendations [13], the forage-to-concentrate ratio being 70:30. Each experimental feed ration was formulated and adjusted to be as isocaloric and isonitrogenous as possible. The average daily allowances of hay, oat and experimental feeds in each dietary treatment are presented in Table 2. About 8% of the oats was substituted with the experimental feeds in the treatments B and C.

The change in rations between periods was made gradually during the first five days of the adaptation period. Feeds were offered in equal meal sizes three times a day at 06:30, 12:30 and 17:30. The grain ration was given about 30 min after the hay ration. The experimental feeds (Feeds 1 and 2) were aimed to be fed at a level of approximately 10% of the total dry matter (DM) intake, the average daily portion being 765 g DM/horse divided into three equal portions that were fed separately after the intake of oats. They were soaked in warm water (45–50 °C) before feeding to ensure their palatability. Mineral intakes were balanced with a commercial vitamin–mineral mixture (Suomen Rehu Ltd., Seinäjoki, Finland).

### 2.3. Feed and Faeces Sampling

Digestibility data were obtained by using chromium mordanted straw (68 mg Cr/g DM) with a daily dose of 1.6 g/kg feed DM as an indigestible external marker for the estimation of apparent digestibility. The chromium mordanted straw was prepared according to Udèn et al. [31]. The chromium dosage was calculated separately for every feed portion and served on the top of the concentrate three times a day, as described in detail by Särkijärvi and Saastamoinen [32]. Samples of hay and oats were collected for analysis over the last seven days of each period, and stored until the end of the five-day collection period. Faecal grab samples (500 g) were taken from each horse twice a day after the morning and mid-day feeding, during the five-day collection period. Samples were collected from the floor of the pen from a freshly produced pile. Daily faecal samples were stored at –24 °C until mixed, sub-sampled and dried (at 100 °C for 1 h + at 60 °C for 72 h) for laboratory analysis [32].

Feed and faeces samples were analysed in the feed laboratory of Luke (Luke Laboratories, Jokioinen, Finland) for dry matter (DM), organic matter (OM), neutral detergent fibre (NDF), crude fibre (CF), crude protein (CP), ether extract (EE) and ash with standard wet chemical methods as described by Särkijärvi et al. [33]. The nitrogen-free extract (NFE) was calculated: (100–CP–CF–EE–ash). The digestible CP (DCP) was calculated: DCP (g/kg DM) = CP (g/kg DM) × CP digestibility (g/kg CP)/1000, where the CP digestibility was taken from the Finnish Feed Tables and Feeding Recommendations [11]. The metabolisable energy value (ME) was calculated according to the British energy evaluation system [34].

### 2.4. Blood Sampling

Blood samples were collected at the end of each period to evaluate the possible effects of the diets on the health of the horses. The samples (2 × 10 mL) were collected 90 min after the morning meal from the jugular vein to heparinised blood collection tubes, and centrifuged. The blood analysis consisted of the contents of red blood cells (RBC), white blood cells (WBC), haemoglobin (Hb), haematocrit (HcT), and fibrinogen, as well as liver enzymes alanine aminotransferase (ALT) and γ-glutamyltransferase (GT), to indicate possible detrimental effects of linseed (cyanogenic glucosides) on the liver. All samples were analysed in the clinical laboratory of Luke.

### 2.5. Statistical Analysis

The data were analysed with a linear mixed model using the MIXED procedure of the SAS system using the REML estimation method. The following statistical model was applied:

Y_ijk_ = μ + a_i_ + t_j_ + p(sq)_k_ + ε_ijk_ (1)

where Y_ijk_ is the observation, μ is the overall mean, a_i_ is the random effect of ith animal (i = 1,…6), t_j_ is the fixed effect of jth dietary treatment (j = 1,…3), p(sq)_k_ is the fixed effect of kth period within the square (k = 1,…3) and ε_ijk_ is the normally distributed error with a mean of 0 and the variance δ^2^. Residuals were tested for normality. The differences were tested with Tukey’s test, and the level of significance was set at the 5% level.

## 3. Results

### 3.1. Feed and Nutrient Intakes

The palatability of all the diets was good and there were no feed refusals. There were only very minor changes (± 0.8–2.2% between the measurements) in the body weights of the horses during the study. Diet intake was isocaloric and isonitrogenous between study periods (all diets combined; Table 3), but the intakes differed between the diets (Table 4). The average intakes of fat (EE) and CP of the supplemented horses (Diets B and C) were 58.0% and 14.1% higher than in the control group. Concerning ME, CF and NDF intakes, the differences were much smaller with +2.0%, –4.0% and –4.5%, respectively.

The proportions of linseed groats (on a DM basis) in diets B and C were 6.7% and 6.3%, respectively. On the BW basis, the intakes of linseed groats were approximately 0.8 g DM/kg BW/d.

### 3.2. Diet Digestibility

The supplementation of the experimental feeds improved only the digestibilities of total diet fat (ether extract, EE) and crude protein (CP) (*p* = 0.0012 and 0.0182, respectively) compared to the control diet (Table 5). In addition, the digestibility of ash (minerals) seemed to be numerically (but not statistically, *p* = 0.2093) somewhat higher in the supplemented diets than in the control diet. None of the digestibility values differed between the supplemental diets (Diet B versus Diet C) (*p*-values = 0.47–0.80).

### 3.3. Blood Parameters

There were no statistically significant differences or trends (*p* > 0.05) in the blood parameters between the diets (Table 6). The variation in the blood parameters (except ALT) was largest when the diet C was fed to the horses. γ -glutamyltransferase (GT) activity was numerically (but not statistically) somewhat higher, and the ALT activity lower, in the linseed supplemented diets. The average number of WBC and concentration of fibrinogen were also numerically higher in linseed supplemented horses but were within the reference values as well. Compared to the other horses, one horse had an exceptionally high GT activity (23–37 U/l), and another individual a low ALT activity (2.0 U/l) during the study period. The Hb and RBC values in all horses were low.

## 4. Discussion

### 4.1. Feed and Nutrient Intakes

Maintaining BW showed that the feeding level used equalled the energy needs [11] of the horses. By period, intakes were isocaloric and isonitrogenous. However, the average fat (EE) intake of the supplemental diets (B and C) was more than 1.5-fold greater compared to the control diet, and the CP was 14.1% higher. In contrast, the differences were minor for energy and fibre components.

The palatability of the studied supplemental feeds was good. In our earlier unpublished study, we observed that the palatability of plain linseed groats was not good when fed in large (more than 6% in DM) portions. Delobel et al. [37] found no effects of flaxseed oil supplementation on diet palatability in horses. The other ingredients (sugar-beet pulp, carrot and molasses) of the linseed supplements fed in the present study likely improved the palatability of the supplemental feeds. Because of the 10% content of dried garlic in the supplemented diets, the horses had to ingest approximately 120 mg/kg BW dried garlic, which has been reported to be within the safe limits of garlic intake given by The National Academies [38].

### 4.2. Diet Digestibility

The improved digestibilities of dietary CP and EE when the supplemental feeds were fed is most likely due to the high concentrations and intakes of those nutrients in the feeds, and is supported by previous studies for CP [32,39,40] and EE [21,41]. In addition, Reitnour and Salsbury [42] found that the caecal administration of linseed meal increased the digestibility of total diet protein. There is, however, no evidence that fat and protein of linseed groats are more digestible than those of the control diet. The improvements may also be partly attributed to the dilution of endogenous faecal nitrogen and fat at higher intakes, which enhances their apparent digestibility [41,43,44,45,46].

Supplementing the diets with the experimental feeds caused a minor decrease in the NDF and CP intakes. The supplemental feeds contained properly digestible fibre sources, carrot and molassed sugar-beet pulp. Both of those ingredients are rich in dietary fibre of a soluble form, and have approximately the same amount of CF, but the NDF content of sugar-beet pulp is approximately two times that of carrot [30,47]. Sugar-beet pulp containing a lot of soluble and highly fermentable fibre [48] has been reported to be well utilised by horses [49,50,51]. No data on the digestibility of carrot is available. In addition, Snel et al. [52] (in their review of studies with different animal species) and Dongowski et al. [53] (in rats) have reported that the dietary fibre in sugar-beet pulp may have a prebiotic effect on intestinal flora improving the microbial activity and, thus, the digestibility. Murray et al. [54] reported in horses that sugar-beet pulp enhanced total diet digestibility fed together with forages. In addition, Lindberg and Palmgren Karlsson [44] explained their results with a positive effect of soluble and fermentable fibre in horses when sugar-beet pulp and fat was added to horse diets. As a potentially contributing factor, Clauss et al. [55] reported that nutrient supply to gut bacteria is the major digestive constraint in horses.

Because the experimental feeds contained many ingredients (being combinations of botanically diverse fibrous feeds), comparisons with previous studies including different diet compositions, and where linseed or variety of its by-products was used, are difficult. It is likely that the other components have their influences too, and that there are confounding and synergistic effects of the diet ingredients. Reitnour and Salsbury [42] found that the caecal administration of linseed meal decreased diet DM digestibility. In our unpublished study, we found that the supplementation of plain linseed groats from 0% to 10% (in the diet DM) gradually decreased the digestibility of the diet nutrients. This has also been reported in dogs [8], and may be due to the poor digestibility of linseed husks and the mucilage content of the hull. Linseed meal is also high in lignin [48], and most of the dietary fibre in linseed meal is in an insoluble form [8]. These findings are supported by Takagi et al. [21] (intakes were not given) who reported very low (21.8) digestibility for the crude fibre of linseed in horses. In one study, the inclusion of extruded linseed (20% in DM) in the diets of horses decreased the digestibility of nutrients compared to hay-only and hay/wheat bran diets [56]. In agreements with the results of the present study, Smolders et al. [57] found increased digestibility values of the diet nutrients when horses were fed compound feed containing (16%) linseed expeller plus more digestible ingredients (cereals). In the present study, the intake of linseed groats was 6.3%–6.7% in DM, and when combined with digestible fibre sources, also improved the diet’s digestibility. Inconsistency of the results between studies is likely due to the different methods and processing of adding linseed or by-products, and different compositions of the diets.

Concerning other animal species, low levels (8%–10% in the feed) of linseed meal in pig feed may improve digestibility and growth rate, but 12% inclusion caused adverse effects [58]. Sled dogs can utilise up to 4.2% linseed cake as a source of fibre without severe reductions in nutrient digestibility or feed consumption [8]. In dairy cows, linseed supplementation improved total tract nutrient utilisation without any adverse effects on ruminal fermentation [59].

Regarding the method applied in determining digestibility, Palmgren Karlsson [60] suggested that chromium mordanted fibre could be an alternative to the administration of chromium, but may result in underestimated digestibility values. In Särkijärvi et al. [33], however, chromium mordanted silage gave quite precise digestibility values in horses (of a similar breed, gender and age as in this study).

### 4.3. Haematology Parameters

The serum concentrations of the liver enzymes alanine aminotransferase (ALT) and γ-glutamyltransferase (GT) were within the reference values of Finnhorses, but close to their lower limit [61]. Elevated ALT and GT values might have indicated possible detrimental effects of linseed (cyanogenic glucosides) on the liver. Additionally, the average number of WBC and concentration of fibrinogen were within the reference values. It can be concluded that there were no inflammatory reactions in the bodies of the horses due to the diet. The large variation in the haematology parameters when the Feed 2 diet was fed to the horses was likely due to the small number of horses used in this study.

The Hb, HcT and RBC values in all horses were low and close to the lower limit of the reference values of Finnhorses [61,62] regardless of the feeding group. The generally low Hb might be a result of the low feeding and exercise intensity of the horses [63].

Based on the blood analyses, we concluded that no adverse health effects in horses were caused from the supplementation of the diet with linseed groat-based feeds (offering approximately 6.3%–6.7% linseed groats in the diet DM or 0.8 g/kg BW/d) during the nine-week experimental period. This is supported by Vineyard et al. [19] who fed milled flaxseed for 70 days without any health problems.

No negative effects on health or performance of sport horses were found when linseed cake (990 g/d) was fed for 60 days [62], which agreed with O’Neil et al. [15] who observed no negative side effects when milled flaxseed was fed (1g/kg BW). The latter researchers concluded that stomach acid can inactivate enzymes within the seeds, which are required to interact with glycosides to form cyanide. Most glycosidases have a pH-optimum of around 7, so in herbivores with acid digestion, e.g., horses, they are usually inactivated [26]. In pigs, Batterham et al. [24] report lighter kidneys, pancreas and spleens for those animals given linseed, but no effects on the weight of livers were observed. They concluded that this may be a result of the anti-nutritional factors of linseed. Mazza and Oomah [26] concluded that most herbivores excrete the unhydrolysed cyanogenic compounds without harm to the animal.

The content of the anti-nutritive compounds in seeds depends on the cultivar, location and year of production, with the cultivar having the most significant effect [6,26]. The current and new *L. usitatissimum* varieties for human nutrition are rather low in toxic and detrimental compounds [26], as were the cultivars used in the present study (Neomed Ltd., Somero, Finland). According to Abraham et al. [63], in case of missing or inactivated glucosidase, the hazard potential (to humans) is low. Boiling is usually recommended to remove the potentially toxic cyanide components, and heat processing of linseed reduces its content of cyanogenic glycosides. Thus, in the present study, both the manufacturing process in the temperatures of 75–80 °C, and soaking into 45–50 °C water before feeding, might also have decreased the content of possible harmful compounds of the linseed groats [6,26]. In addition, HCN content is reduced when linseed is mixed with several ingredients and when the product is pelleted [64] as in the present study.

## 5. Conclusions

Linseed groat-based supplements (offering approximately 6.3%–6.7% linseed groats in the diet DM or 0.8 g/kg BW/d), and containing soluble fibre sources (sugar-beet pulp, dehydrated carrot), improved the crude protein and fat digestibility of hay/oats diets of horses, and had no effects on fibre digestibility. No adverse or anti-nutritional effects were observed on the availability of any component of the diet or the haematologic parameters and health of the horses. Linseed by-products combined with other fibre sources can be used, for example, in feeding strategies replacing grains in the horse rations in order to reduce the intake of starch. There is a need to investigate the synergetic and confounding effects of diet ingredients of different sources, especially botanically diverse fibrous feeds.

## Figures and Tables

**Table 1:** Average chemical composition of the experimental feeds (g/kg dry matter).

Composition	Hay	Oats	Feed 1	Feed 2
Dry matter g kg^-1^	870.7	883.1	905.2	885.4
Organic matter	934.7	971.4	930.6	924.3
Crude protein	95.0	112.8	211.5	209.8
Ether extract	15.6	61.0	185.7	172.7
NDF	687.7	263.3	183.3	228.0
Crude fibre	339.0	89.0	96.0	105.3
Ash	63.5	28.6	69.4	75.7
NFE	485.1	708.6	437.5	436.5
ME MJ/kg DM	9.10	12.6	14.3	13.9

NDF = neutral detergent fibre; NFE = nitrogen free extract; ME = metabolisable energy.

**Table 2:** Daily allowances of hay, oats and experimental feeds (Feed 1 or Feed 2) fed in each dietary treatment (DM kg/day) (with ranges).

Feed	Diet A (Control)	Diet B (Feed 1)	Diet C (Feed 2)
**Hay**	5.89 (5.66–6.36)	5.44 (5.22–5.92)	5.43 (5.22–5.75)
**Oats**	1.85 (1.80–2.12)	1.74 (1.68–1.89)	1.74 (1.68–1.84)
**Experimental feed**	—	0.757 (0.710–0.780)	0.745 (0.730–0.750)

**Table 3:** Average daily ME, CP and DM intakes (± s.d.) for each period.

Intake	Period I	Period II	Period III
**ME MJ**	80.3 ± 4.8	81.8 ± 3.9	80.1 ± 3.2
**CP g**	832.4 ± 68.9	850.4 ± 73.1	835.2 ± 50.6
**DM kg**	7.86 ± 0.37	7.98 ± 0.29	7.86 ± 0.31

ME = metabolisable energy; CP = crude protein; DM = dry matter.

**Table 4:** Average daily dry matter, metabolisable energy and nutrient intakes (±s.d.) in each dietary treatment.

Intake	Diet A (Control)	Diet B (Feed 1)	Diet C (Feed 2)
**DM kg**	7.77 ± 0.39	7.94 ± 0.28	7.91 ± 0.27
**ME MJ**	77.3 ± 3.9	82.9 ± 3.0	81.5 ± 2.6
**EE g**	206.4 ± 10.8	332.8 ± 9.7	318.9 ± 6.5
**CP g**	763.9 ± 40.2	877.5 ± 29.6	867.3 ± 26.5
**DCP g**	518.5 ± 26.5	618.2 ± 18.2	612.0 ± 23.2
**NDF g**	4556.3 ± 209.1	4338.6 ± 156.8	4358.9 ± 166.6
**CF**	2164.8 ± 108.0	2072.0 ± 74.4	2073.9 ± 83.0

DM = dry matter; ME = metabolisable energy; EE = ether extract (fat); DCP = digestible crude protein; CP = crude protein; NDF = neural detergent fibre; CF = crude fibre.

**Table 5:** Average apparent digestibility coefficients (%) and standard deviations (s.d.) for the total diet nutrients in the control and experimental diets.

Composition	Diet A (Control)	Diet B	Diet C	*p* Value
		(Feed 1)	(Feed 2)	(B and C vs. Control Diet)
Dry matter	54.8 (5.29)	55.3 (2.54)	57.0 (3.96)	0.5065
Organic matter	56.9 (5.05)	57.2 (3.24)	58.9 (3.92)	0.5710
Crude protein	61.4 (6.20)	64.0 (4.46)	65.7 (5.83)	0.0182
Ether extract	56.2 (7.99)	68.0 (5.02)	68.8 (5.46)	0.0012
NDF	47.2 (6.01)	47.8 (4.17)	47.9 (4.16)	0.8139
Crude fibre	44.6 (5.27)	43.9(4.45)	46.3 (4.87)	0.8484
Ash	19.7 (20.08)	25.1 (8.74)	27.4 (10.68)	0.2093
NFE	62.8 (5.09)	62.0 (2.83)	63.5 (3.32)	0.9601

NDF = neutral detergent fibre; NFE = nitrogen free extract.

**Table 6:** Average values of blood parameters and standard deviations (s.d.) for the horses in the control and experimental diets.

Blood Parameters	Diet A (Control)	Diet B (Feed 1)	Diet C (Feed 2)	Reference Values [35,36]
Glutamyltransferase, U/l	17.0 (4.46)	19.7 (6.66)	18.0 (7.10)	10–70
Alanine aminotransferase, U/l	6.33 (2.02)	5.67 (2.57)	5.83 (1.95)	5–45
Haemoglobin, g/l	127.7 (7.27)	124.2 (5.64)	126.0 (11.03)	120–155
Haematocrit, %	34.7 (2.15)	34.3 (1.88)	34.4 (3.51)	34–43
Red blood cells, ×10^12^/l	6.99 (0.55)	6.86 (0.55)	6.92 (0.89)	7.0–9.0
White blood cells, × 10^9^/l	7.35 (1.02)	7.25 (0.69)	7.23 (1.72)	4.6–9.5
Fibrinogen, g/l	2.67 (0.28)	2.78 (0.25)	2.86 (0.34)	1.2–4.0

None of the differences between the groups were statistically significant (all *p* > 0.05; *p*-values 0.6–1.0).
